# Population differentiated copy number variation between Eurasian wild boar and domesticated pig populations

**DOI:** 10.1038/s41598-022-22373-z

**Published:** 2023-01-20

**Authors:** Jisung Jang, Bongsang Kim, So Yun Jhang, Byeongyong Ahn, Mingue Kang, Chankyu Park, Eun Seok Cho, Young-Sin Kim, Woncheoul Park, Heebal Kim

**Affiliations:** 1 Interdisciplinary Program in Bioinformatics, Seoul National University, Seoul, Republic of Korea; 2 eGnome, Inc, Seoul, Republic of Korea; 3 Department of Agricultural Biotechnology and Research Institute of Agriculture and Life Sciences, Seoul National University, Seoul, Republic of Korea; 4 Department of Stem Cell and Regenerative Biotechnology, Konkuk University, Seoul, 05029 Korea; 5 Swine Science Division, Rural Development Administration, National Institute of Animal Science, Cheonan, South Korea; 6 Animal Genomics and Bioinformatics Division, National Institute of Animal Science, RDA, Wanju, 55365 Republic of Korea

**Keywords:** Evolutionary genetics, Population genetics

## Abstract

*Sus scrofa* is a globally distributed livestock species that still maintains two different ways of life: wild and domesticated. Herein, we detected copy number variation (CNV) of 328 animals using short read alignment on Sscrofa11.1. We compared CNV among five groups of porcine populations: Asian domesticated (AD), European domesticated (ED), Asian wild (AW), European wild (EW), and Near Eastern wild (NEW). In total, 21,673 genes were identified on 154,872 copy number variation region (CNVR). Differences in gene copy numbers between populations were measured by considering the variance-based value ${V}_{ST}$ and the one-way ANOVA test followed by *Scheffe* test. As a result, 111 genes were suggested as copy number variable genes. Abnormally gained copy number on *EEA1* in all populations was suggested the presence of minor CNV in the reference genome assembly, Sscrofa11.1. Copy number variable genes were related to meat quality, immune response, and reproduction traits. Hierarchical clustering of all individuals and mean pairwise ${V}_{ST}$ in breed level were visualized genetic relationship of 328 individuals and 56 populations separately. Our findings have shown how the complex history of pig evolution appears in genome-wide CNV of various populations with different regions and lifestyles.

## Introduction

Pig (*Sus scrofa*) is by far one of the most globally distributed animal species maintaining two different ways of life: wild and domesticated. The great adaptability of wild boar makes it possible to colonize the wild areas, including mainland Eurasia and North Africa, within 2 Mya, after originating from Southeast Asia in the early Pliocene 5.3–3.5 Myr ago^1^. In addition to adaptation to various environments of the wide habitats, demographic events such as migration and bottleneck during the glacial periods also make pigs diverge into numerous populations. The two main populations of wild boar, European and Asian, diverged around 1 Mya^2^. Initial domestication took place independently at two locations, East Anatolia and China with local wild boars in 9000 to 10,000 years ago^3^. Mitochondrial DNA analysis by (mtDNA) suggested that European domesticated pigs arrived from Near East alongside farmers 8500 YBP^4^.

The population and geographical distribution of the domesticated pigs have greatly varied from wild boars after initial domestication because of long-term climate fluctuations, human hunting, and follow-up stock-raising activities^5^. However, domesticated pigs and wild boars were not only consistently diverged from one another. For instance, over 3000 years after the arrival of Near Eastern domesticated pigs to Europe, domesticated pigs were interbred with local wild boar. It made most of Near Eastern ancestry disappear in the genomes of European domesticated pigs^4,6^. Subsequent selection and breeding of domesticated pigs resulted in highly distinct pig breeds in Europe and Asia^7^. Domesticated pigs have undergone a complex history of selection and migration to improve commercial traits. For example, European farmers induced introgression between Asian and European domesticated pigs to improve commercial traits such as litter size and backfat in the early nineteenth century^8^. Modern breeding practices, including reproductive isolation and genomic selection, have accelerated genetic divergence between wild boar and domesticated pigs since the foundation of modern pig breeds, starting around 200 years ago. Previous genome-wide SNP studies identified distinct patterns of selection in domesticated pigs and wild boars^9,10^.

Copy number variation (CNV) is another type of variation which covers more significant part of the porcine genome than single nucleotide polymorphism (SNP). CNV can be a major mechanism driving genome evolution, especially in gene expression. Generally, CNVs are more recent events than SNPs as they are still segregating within the population, showing 2.5 times faster evolution rate in the porcine genome^6^. Copy number variable genes in the porcine genome were suggested as candidates for selection related to traits such as coat color^11,12^, backfat thickness^13^, fatty acid composition, growth^14^, and reproduction^15^. Therefore, comparing CNV can be an effective strategy for identifying recently accelerated differentiation between wild boar and domesticated pigs.

However, the number of individuals and populations in most of previous studies was not enough to suggest differentiated genomic regions between pig populations, such as indigenous breeds and wild boars. Furthermore, the credibility and resolution of CNVs were limited by using SNP chip or aligning on an older version of genome assembly. Here, we defined porcine CNVs from 328 individuals in 56 breeds, the largest population that represents their CNVs, including wild boar and domesticated and indigenous populations from broad area in Europe and Asia. We expected that our study on the comparison of pig CNVs between domesticated and wild would improve further understanding of the evolution of *Sus scrofa*.

## Methods

### Sample collection

The study population consisted of 328 individuals consist of 130 females and 198 males from 56 pig populations. The whole-genome sequencing (WGS) of wild boar and domesticated breeds were collected from Europe and Asia. 313 genomes were publicly available and sequenced using Illumina paired-end library and from SRA database (Table S1). 15 genomes including 5 Duroc, 5 Woori-Heukdon and 5 Korean Native were newly sequenced in this study. The 15 Blood samples were collected during routine veterinary treatments with the logistical support under the ethical approval of National Institute of Animal Science, Republic of Korea (NIAS20212224). All of the experimental protocols were approved by National Institute of Animal Science, Republic of Korea (NIAS20212224).

The 56 *Sus scrofa* populations were classified into five groups, European domesticated (ED), Asian domesticated (AD), European Wild Boar (EW), Asian Wild (AW), and Near Eastern Wild (NEW) as follows: (i) 109 individuals of ED including 1 Angler Sattelschwein, 11 Berkshire, 1 British Saddleback, 1 Bunte Bentheimer, 2 Casertana, 1 Chato Murciano, 17 Duroc, 1 Gloucester Old Spot, 3 Hampshire, 4 Iberian, 4 Landrace, 37 Large White (Yorkshire), 2 Leping Spotted, 1 Linderodsvin, 5 Mangalica, 2 Middle White, 1 Nero Siciliano, 13 Pietrain and 2 Tamworth; (ii) 120 individuals of AD including 6 Bamaxiang, 7 Bamei, 6 Baoshan, 3 Enshi black, 21 Erhualian, 6 Hetao, 3 Jiangquhai, 9 Jinhua, 5 Korean native, 5 Woori-Heukdon, 6 Laiwu, 6 Luchuan, 10 Meishan, 6 Min, 7 Neijiang, 6 Rongchang, 3 Tongcheng, 2 Wannan Spotted, 2 Xiang, and 1 Zang; (iii) 20 individuals of EW including 12 Dutch, 1 French, 4 Italian, 2 Spanish and 1 Swiss wild boar; (iv) 77 individuals of AW including 65 Chinese, 1 Japanese, 10 Korean, and 1 Russian wild boar; (v) 2 Near Eastern wild boar. The additional information of samples is described in Table S1.

### Whole genome sequencing

Fifteen genomes including 5 Duroc, 5 Woori-Heukdon and 5 Korean Native were newly sequenced in this study. Blood samples were collected for DNA extraction by Wizard® Genomic DNA Purification Kit (Promega) from National Institute of Animal Science, Rural National Institute of Animal Science, Republic of Korea. Library construction was performed for each individual using 2 μg of genomic DNA with Illumina TruSeq PCR-free (550) Kit. Sequencing was performed to generate 2 × 151 paired-end reads on the Illumina NovaSeq 6000 platform.

### Whole genome sequence alignment

After quality control checking of raw reads using FastQC-0.11.8^16^, adapter and low-quality bases of reads were trimmed by Trimmomatic-0.39^17^. After checking the trimming results and quality of trimmed reads, the trimmed reads were mapped using BWA-0.7.17 MEM^18^ to reference genome Sscrofa11.1 assembly^19^. The outputs of the sequence alignment map (SAM) were sorted, indexed, and compressed to binary format (BAM) by Samtools-1.9^20^. The duplicates in BAM files were marked using Picard 2.20.2 MarkDuplicates (https://broadinstitute.github.io/picard/), and the marked BAM files were used as input for variant calling. The alignment rate, coverage, and mean depth were calculated using Sambamba^21^.

### CNV, CNVR and candidate of differentiated gene definition

A combination of the CNVnator v0.4.1^22^ and LUMPY v0.3.1^23^ software was used to identify putative CNV of porcine genomes. CNVnator is a read depth method while LUMPY uses discordant alignment such as split reads and paired-end mapping. CNVs of all samples were called with a bin size of 200 bp by CNVnator and filtered with size (> 1 kb), p-value calculated using t-test statistics (< 0.001) and fraction of reads with zero mapping quality (MQ0 < 0.5). The CNVs in unplaced scaffolds were removed. Structural variations including CNV were detected by ‘lumpyexpress’ command of LUMPY with default parameter^23^. Overlapped copy number variable regions with same type of CNV between results of CNVnator and LUMPY were defined as concordant CNVs in every individual. The chromosomal distribution of the concordant CNVs were compared between male and female, p-arm and q-arm, and among populations. A 50% reciprocal overlap between filtered CNVs was defined as copy number variation region (CNVR) using CNVRuler^24^. CNVRs found in two and more of individuals were used for downstream analysis to minimize false-positive. Copy number of every gene on CNVR were calculated based on aligned read depth and normalized using CNVnator^22^. The normalized copy number of neutral region from diploid autosome was assumed to be 2.0.

### Hierarchical clustering based on CNVR

To cluster individuals according to their CNV similarities, we made a vector representing presence or absence of CNV for each individual of genes on CNVRs. Hierarchical clustering with 1000 bootstrap resampling was performed on these vectors for genes on autosomal CNVR using *pvclust* with the default option in R^25^. The ‘correlation’ and ‘average’ were used as distance measures and the agglomerative method, respectively. The approximately unbiased (AU) *p*-value was calculated by multiscale bootstrap resampling. The bootstrap probability (BP) *p*-value was calculated by ordinary bootstrap resampling based on the unweighted pair-group average method (UPGMA).

### Copy number variable genes between populations

The normalized copy number of genes on CNVRs of all individuals was calculated using CNVnator^22^. The normalized copy number of the neutral region from diploid autosome was assumed to be 2.0. ${V}_{ST}$ of normalized copy number between a pair of populations was calculated as ${V}_{ST}$ = (${V}_{T}$ −  ${V}_{S}$)/${V}_{T}$, where ${V}_{T}$ is the total variance of normalized copy number among all individuals from both populations, and ${V}_{S}$ is the average of variance within each population, weighted by the number of individuals in the population^26^. After excluding the ten populations (ANG, BRI, BUN, GLO, LIN, NES, WJP, WRU, WSW, ZAN) with a single animal ${V}_{ST}$ between pairs of 56 *Sus scrofa* populations were calculated. Mean ${V}_{ST}$ of all genes on autosomal CNVRs in each pair of breeds were visualized using *pheatmap* in R^27^. In addition, the ${V}_{ST}$ of autosomal copy number variable genes were calculated between AD, AW, ED, EW, and NEW. These results were visualized as Manhattan plots using *qqman* package in R^28^.

One-way ANOVA test on copy number of every genes on autosomal CNVRs were performed on 5 groups including AD, AW, ED, EW, and NEW. As a post hoc test of ANOVA, *Scheffe* test was performed on genes of which ANOVA resulting *p*-values was smaller than 0.05. Genes on CNVR which satisfy both upper 1% pairwise ${V}_{ST}$ and the *p*-value less than 0.05 of *Scheffe* test after one-way ANOVA were defined as population differentiated genes. Hypothetical, putative, predicted, or uncharacterized genes, as well as pseudo-genes, were excluded.

### Ethics approval and consent to participate

The 15 Blood samples (5 Korean Native Pigs, 5 Woori-Heukdon, and 5 Duroc individuals) were collected during routine veterinary treatments with the logistical support under the approval of NIAS20212224, Republic of Korea. No further ethics permissions were required for this study. All animals were handled in strict accordance with good animal practice.

## Results

### Sequence alignment, CNV calling and CNVR definition

**Figure 1:**
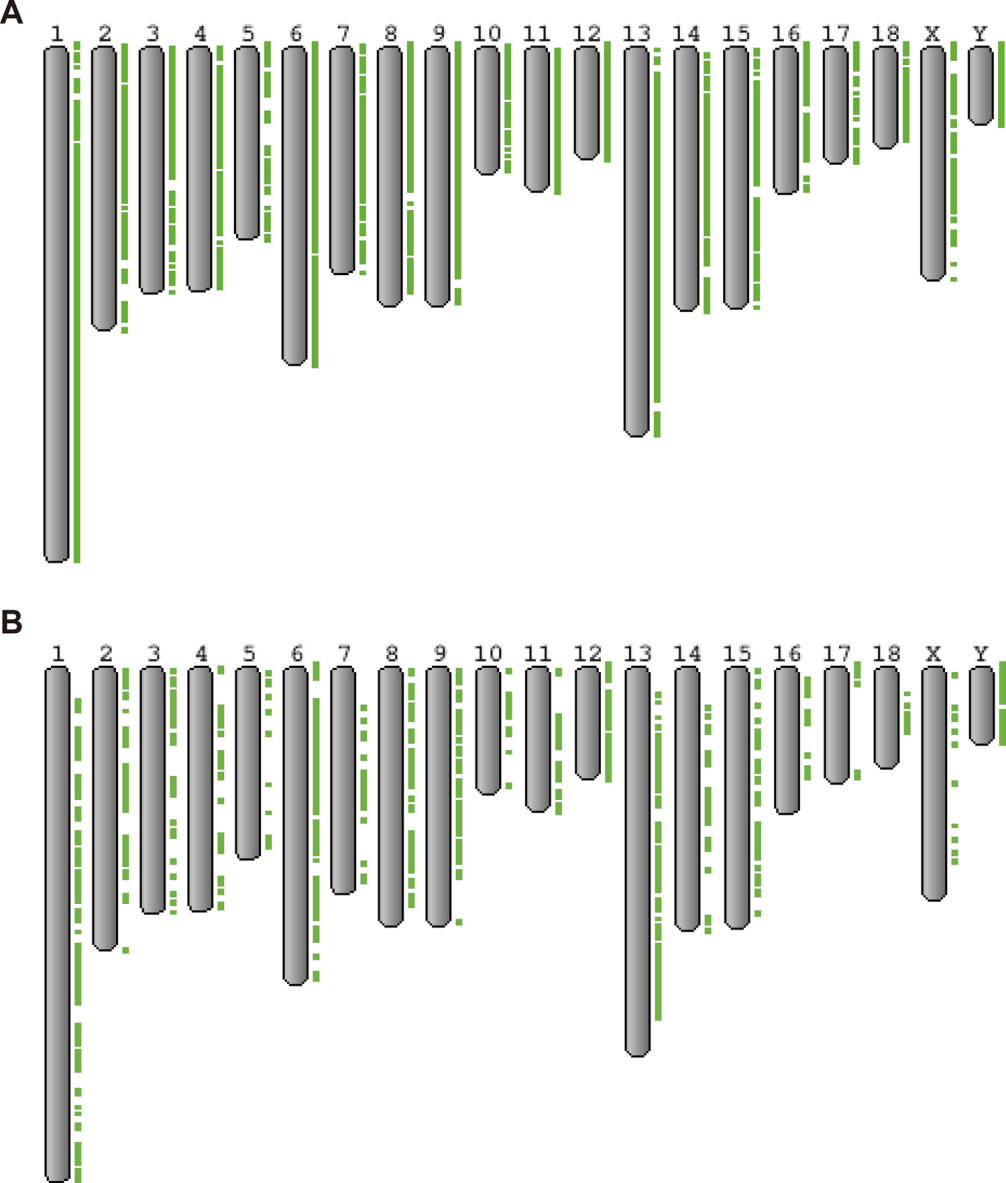
CNVR distribution. Distribution of CNVRs larger than 100 kb (**A**) and 500 kb (**B**) were visualized separately. Green rectangles on the right side of chromosomes represents CNVRs.

The coverage and sequencing depth are important for the credibility of CNVs called using the read depth information of short read alignment. Sequence alignment statistics including mapping rate, coverage and mean depth of all samples were summarized in Table S1. In our dataset, the minimum mean depth was higher than 5.06×, and the mean values of alignment rate, coverage, and mean depth of coverage were about 99.3%, 96.4%, and 18.5×, respectively (Table S1). Number of CNVs defined by CNVnator, Lumpy and consensus CNV of the two software was summarized in Table S2. Lumpy called more CNVs especially deletion than CNVnator in most of individuals. After calling and filtering CNVs, genome-wide CNVRs were identified. Chromosome-wise distribution of CNVs and their total length was summarized in Table S3. Among chromosomes, the ratio of total length of CNV to chromosome size were the largest in chromosome Y, followed by chromosome 12 and 6 while the smallest in chromosome 18 followed by 16 and 15. Total length of CNVs were larger in female than male in chromosome 12 and 2, while smaller in chromosome 11, and 16 (Table S4). CNV distribution on p-arm and q-arm were also compared based on centromeric region defined in the reference genome. Most of centromere-defined chromosome had more CNVs on q arm while less CNVs on q arm in chromosome 3, 5 and 9 (Table S5). Distribution of CNVR larger than 100 kb and 500 kb were visualized separately in Fig. 1. Average size of autosomal CNV of AD, AW, ED, EW and NEW were about 51.7, 51.9, 37.9, 26.6 and 9.0 Mbp. Average lengthening and shortening of chromosomal length in each group was summarized in Table S6. There were population specific lengthening and shortening of chromosomal length in chromosome 4–6, 8 and 14–18 (Table S6). All genes on CNVRs with statistics were summarized in Table S7.

### Population differentiation based on copy number variable genes

**Figure 2:**
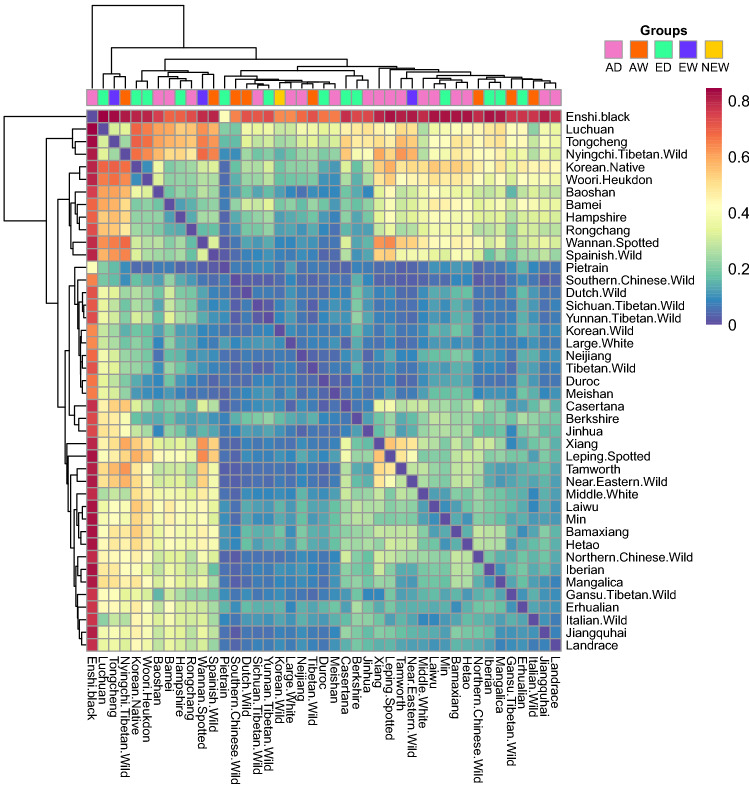
Average of pairwise ${V}_{ST}$ between breeds. Average of pairwise ${V}_{ST}$ of genes on autosomal CNVRs were calculated between all pairs of breeds which included more than 1 sample. Clustering were performed only on the mean pairwise ${V}_{ST}$.

Hierarchical clustering of all individuals was performed on vectors considering the presence or absence of autosomal CNVRs (Fig. S1). Mean pairwise ${V}_{ST}$ values of breeds including more than one animal were calculated on 315 animals from 43 populations and visualized as a heatmap with hierarchical clustering (Fig. 2). The ${V}_{ST}$ range is from 0 to 1, with a higher value indicating a larger difference. The pairwise mean ${V}_{ST}$ values of five groups were as following: AD-ED, 0.009; AD-AW, 0.032; AD-EW, 0.015; AD-NEW, 0.005; ED-EW, 0.012; ED-AW, 0.040; ED-NEW, 0.005; AW-EW, 0.020; AW-NEW, 0.007; EW-NEW, 0.020. The average of the pairwise ${V}_{ST}$ in groups was about 0.017, and the average in breed level was 0.240.

### Copy number variable genes across populations

**Figure 3:**
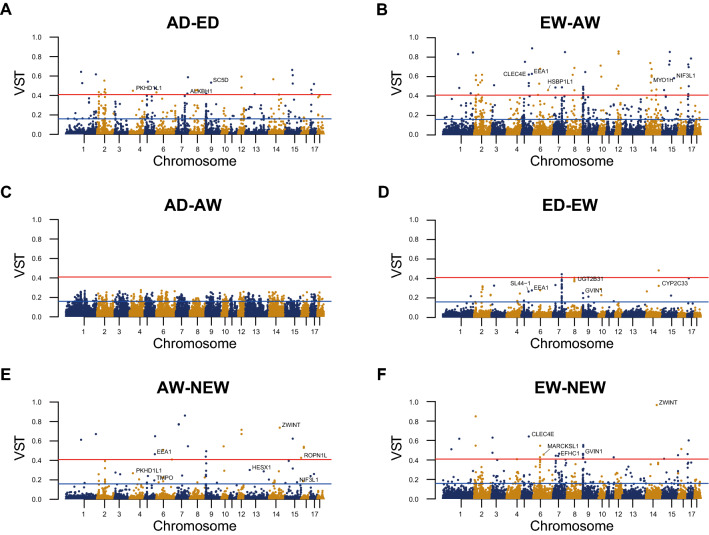
Manhattan plot of ${V}_{ST}$. ${V}_{ST}$ of genes on autosomal CNVRs were visualized as Manhattan plots. The center point of genes was used as an x-coordinate value. Genes with significantly different pairwise ${V}_{ST}$ in upper 0.1% were marked by their names. Name of hypothetical, putative, predicted or uncharacterized genes and pseudo-genes were excluded due to lack of space. The upper 1% percentile ${V}_{ST}$, 0.157, and upper 0.1% percentile, 0. 409, were shown as blue and red lines, respectively.

Candidates of copy number variable genes were suggested based on the two criteria; pairwise ${V}_{ST}$ and *Kruskal–Wallis test* across five groups, including AD, ED, AW, EW, and NEW. First, ${V}_{ST}$ was calculated between pairs of five groups. The upper 1% and upper 0.1% values of pairwise ${V}_{ST}$ between groups were about 0.159 and 0.409, respectively. Pairwise ${V}_{ST}$ of genes on autosomal CNVR were visualized as Manhattan plot (Fig. 3). There were some peaks shared by pairs of groups. We suggested the shared peaks between pairs including a same group as the regions with copy numbers distinct from other groups.

**Figure 4:**
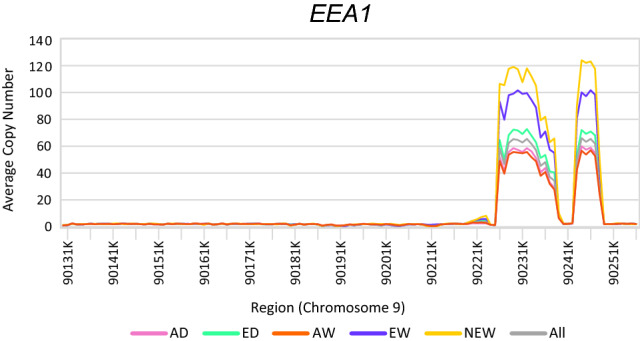
Average copy number of 5 groups in *EEA1*. Average copy number around *EEA1* coding region. X-axis indicated genomic region and y-axis indicated average copy number in each group. *EEA1* located from 90,131,707 to 90,257,014 in chromosome 5 and the average copy number of every 1000bp regions from 90,131,001 to 90,258,000 were visualized as a line graph. The two peak regions were 90,227,001–90,240,000 and 90,244,001–90250000.

Then, differences of normalized copy numbers across the five groups were tested using the one-way ANOVA followed by *Scheffe* test. Among genes of which the *p*-value was below 0.05, 111 genes of which ${V}_{ST}$ values in the upper 0.1% of at least a pair of groups defined as copy number variable genes (Table S7). 15 genes were remained after excluding hypothetical, putative, predicted, or uncharacterized genes, as well as pseudo-genes (Table 1). Among these copy number differentiated genes, group-wise average copy number of every 1 kb of *EEA1* were visualized in Fig. 4.

**Table 1:** Genes with differentiated copy number between populations.

Gene	Chromosome	Start	End	ANOVA F value	ANOVA p-value	Scheffe p-value	VST upper 0.1% pair	Average copy number				
								AD	ED	AW	EW	NEW
PKHD1L1	4	28,023,448	28,176,331	67.120	2.74.E−41	AD-ED, AD-EW, AD-NEW, AW-ED, AW-EW, AW-NEW	AD-ED	1.50	1.54	1.81	1.78	2.02
CLEC4E	5	63,219,229	63,228,566	69.530	2.05.E−42	AD-ED, AD-EW, AW-ED, AW-EW, ED-EW	AW-ED, AD-EW, AW-EW, EW-NEW	0.94	0.91	1.55	1.82	1.01
EEA1	5	90,131,707	90,257,014	60.350	5.01.E−38	AD-ED, AD-EW, AD-NEW, AW-ED, AW-EW, AW-NEW, ED-EW, ED-NEW	AD-EW, AW-EW, AW-NEW	9.06	8.83	10.90	14.75	17.27
MARCKSL1	6	88,785,412	88,787,772	18.560	9.60.E−14	AD-ED, AD-EW, AW-ED, AW-EW	EW-NEW	1.88	1.67	1.25	1.14	2.34
HSBP1L1	6	127,960,330	127,972,241	38.980	1.26.E−26	AD-ED, AD-EW, AW-ED, AW-EW, AW-NEW	AW-EW	2.64	2.69	2.30	2.09	2.02
EFHC1	7	46,244,915	46,320,261	5.790	1.64.E−04	AD-NEW, AW-ED, ED-NEW, EW-NEW	EW-NEW	2.03	2.09	2.03	2.03	2.37
ALKBH1	7	100,676,778	100,710,414	57.500	1.32.E−36	AD-ED, AD-EW, AW-ED, AW-EW	AD-ED	1.91	1.94	2.10	2.14	1.97
UGT2B31	8	66,310,697	66,323,755	27.970	5.87.E−20	AD-AW, AD-ED, AD-EW, AD-NEW, AW-EW, AW-NEW, ED-EW, ED-NEW	AD-EW	2.64	3.07	3.06	4.52	5.03
GVIN1	9	2,874,233	2,882,380	6.720	3.34.E−05	AD-EW, ED-EW	EW-NEW	1.10	1.29	1.04	1.76	0.39
SC5D	9	48,357,115	48,372,391	88.640	9.30.E−51	AD-ED, AD-EW, AW-ED, AW-EW	AD-ED, AW-ED	1.97	2.00	2.36	2.29	2.00
MYO1H	14	41,469,191	41,588,934	41.450	4.85.E−28	AD-ED, AD-EW, AW-ED, AW-EW, ED-EW	AW-EW	1.70	1.76	1.55	1.45	1.45
ZWINT	14	94,094,781	94,109,447	65.47	1.66.E−40	AD-NEW, AW-NEW, ED-NEW, EW-NEW	ED-NEW, AD-NEW, EW-NEW, AW-NEW	2.11	2.15	2.13	2.20	4.06
CYP2C36	14	106,184,665	106,219,631	40.27	2.29.E−27	AD-ED, AD-EW, AW-ED, AW-EW, ED-NEW	AW-ED	3.41	3.65	2.22	2.50	4.38
NIF3L1	15	104,583,736	104,606,507	25.3	3.01.E−18	AD-ED, AD-EW, AW-ED, AW-EW, ED-EW	AD-EW, AW-EW	1.74	1.78	1.98	2.34	2.15
ROPN1L	16	43,299	58,569	9.78	1.76.E−07	AD-NEW, AW-NEW, ED-NEW, EW-NEW	AW-NEW	1.86	1.82	1.88	2.04	3.01

## Discussion

Since the colonization of wild boar across mainland Eurasia and North Africa within two Mya and domestication started 10,000 years ago, *Sus scrofa* has been adapted to various environments and human needs. In addition to selection pressure, demographic events such as the bottleneck in the last glacial period about 20,000 years ago and migration following farmers intensified the development of various pig breeds. Furthermore, modern breeding programs have accelerated genomic studies on pigs with the aim of improving their value as a source of meat and model animals. In particular, porcine CNV has been a great subject for studying phenotypic variance, especially in quantitative traits, as it can alter gene dose and expression. Our study analyzed the largest number of Eurasian wild boar and domesticated pigs with two values to measure the differences in copy number between populations. The first was ${V}_{ST}$ based on variance, and the second was the one-way ANOVA test. Considering both values together, we present the copy number variable regions and compare the copy number between populations. Chromosome-wise distribution of CNVs were compared by population, sex and chromosomal location such as p-arm and q arm separately. The autosomal CNVs covered larger regions in Asian pigs than European pigs which might be results of reference bias of using single reference representing Duroc. On the other hand, we could not observe any consistent effects of sex and chromosomal location on prevalence of CNVs. There would be other multiple genomic features which affect the probability of CNV occurrence.

Hierarchical clustering was performed on vectors representing the presence or absence of CNVs on autosomal CNVRs. Some individuals were clustered following their groups while others were not. For example, Pietrain individuals were clustered discordant with their breeds. Actually, variance of copy numbers was highest in Pietrain among breeds with the value (1.06) significantly higher than others, followed by the variance of Meishan (0.33). Thus, both the clustering result and the high variance of copy numbers indicate that the within-variance of Pietrain is higher than other breeds.

Whether domesticated or in the wild, most individuals were clustered along their region rather than their way of life. It implies that gene flow between domesticated pigs and wild boar is still occurring in some areas. Even with the separation between domesticated and wild, the impact of artificial selection on porcine CNV may not be large enough to surpass the impact of gene flow between domesticated and wild.

All the Woori-Heukdon (KWH) and Korean native pigs (KNP) were clustered together with Duroc. KWH was developed by crossbreeding of three generations starting from pure Duroc sow and KNP, also called Chookjin-Chamdon. The F1 hybrid sow was crossed with pure Duroc boar, and the F2 hybrid sow was crossed with Duroc boar. Because the breed development was a recent event finished in 2011, the inherited CNV of KWB has been changed a little.

Since the pairwise ${V}_{ST}$ becomes smaller when ${V}_{S}$ becomes larger, almost all pairs of breeds with Pietrain of which the variances in copy number was the largest one, had the smallest ${V}_{ST}$. In contrast, all pairs with Enshi black pig had the highest ${V}_{ST}$. Due to the fact that the distance between breeds in clustering on the heatmap was only measured with the mean value of pairwise ${V}_{ST}$, the clustering of breeds was not always concordant with their groups.

Copy number alteration of genes can make drastic change in phenotype by affecting on the expression and the structure of protein. Therefore, the copy number differentiated genes would be suggested as candidate regions of selection. We suggested how CNVs involved in the evolution of each population by considering environmental differences between respective population and functions of copy number differentiated genes.

Polycystic Kidney and Hepatic Disease 1-Like 1 (*PKHD1L1*) encodes a member of the polycystin protein family containing 11 transmembrane domains. *PKHD1L1* has been reported as a candidate gene for variation in pH of pork^29^, which is related to meat color and water holding capacity. The average copy numbers of *PKHD1L1* were slightly lost in groups except for NEW, and they were slightly higher in the European than Asian population. This CNV would be a causative variation on the difference in meat color and water holding capacity between populations.

CLEC4E encodes C-type lectin domain family 4 member E protein. The protein, also called Mincle (Macrophage inducible C-type lectin), is an innate immune receptor on myeloid cells sensing pathogens^30^. Since it was first described as a receptor for mycobacterial cell wall glycolipid and cord factor, the role of Mincle in innate immunity against mycobacterial infection has been investigated. Upregulation of Mincle expression in response to mycobacterial infection were observed in mice^31^. When Mincle senses the motif of microbial signal, it induces pro-inflammatory responses. In addition to this fundamental role as a receptor, Mincle can act as an immune modulator in different models by either promoting anti-inflammatory cytokines expression or downregulating pro-inflammatory signaling pathways^30,32^. Tuberculosis, mainly caused by mycobacterial infection, is a severe threat to pigs. Wild boar was suggested as a reservoir that maintains and spreads tuberculosis infection^33^. The copy numbers of *CLEC4E* were lost in domestic groups and NEW while neutral in EW and AW. The higher copy number of the CLEC4E in wild boars may be presented as evidence of adaptation to mycobacterial infection prevalent in the wild environment.

The average copy number of early endosome antigen 1 encoding gene, *EEA1* in every groups was more than 8.8 (Table 1). These abnormal copy numbers are most likely caused by minor variations in the reference genome. We demonstrated average copy numbers of five groups in genomic regions, including upstream, protein coding, and downstream region of *EEA1* in Fig. 3. The average copy numbers in all groups peaked in two regions: 90,227,001–90,240,000 and 90,244,001–90,250,000. Furthermore, the homologous shape of the graphs among all groups also supported the possibility of minor deletion in the reference genome. *EEA1* consists of 5′ upstream, 31 exons, 30 introns, and 3′ downstream sequences, and the peak regions covered exons 16–21, 23, 24 and their adjacent introns. The previous gene reconstruction using additional alternate transcripts of pig individuals also improved a model of *EEA1* whose model was missed in Ensembl^34^.

The *GVIN1*, interferon-induced very large GTPase 1, was upregulated in *PRRSV*-infected porcine alveolar macrophage^35^ while downregulated in lungs during bacterial respiratory infection^36^. However, the biological mechanism of *GVIN1* expression against infection and the phenotypical effect of deletion in the porcine genome remain poorly understood.

Kojima and Degawa^37^ demonstrated that *UGT2B31* expression was higher in male pigs when compared to female pigs and that testosterone treatment of castrated boars increased *UGT2B31* expression. Considering the above literature and gene expression network, Sahadevan et al.^38^ suggested that *UGT2B31* could play steroid metabolic roles in porcine androgen/androstenone metabolism. Sabmborski et al.^39^ also demonstrated a significant decrease in *UGT2B31* expression on day 14 of the pregnant pig. These previous studies continuously identified the role of *UGT2B31* in steroid hormone biosynthesis. The copy number of *UGT2B31* in EW and NEW groups were significantly gained. Moreover, *SC5D* is another gene involved in steroid biosynthesis, such that the expression of *SC5D* was upregulated in the pig ovary during the luteal phase^40^. The copy number of *SC5D* was significantly different in our rank- and variance-based test, and the average copy numbers were slightly higher in European pigs than in others. Therefore, these steroid syntheses related genes could be suggested as candidates which can make a difference in reproductive traits between porcine populations.

Cytochrome P450 (CYP) is a type of oxygenase. A previous study identified differences in the fatty acid composition of adipose tissues between Korean native and Yorkshire pigs^41^. The significantly higher expression of CYP genes in Yorkshire was presented as the cause of lower arachidonic acid and higher cis-11,14,17-Eicosatrienoic acid, which are responsible for meat flavor. One of CYP isoforms *CYP2C36* was also suggested as copy number variable genes in our result. The mRNA levels of *CYP2C33*, *CYP2C49*, *CYP3A29,* and *CYP3A46* were reported as significantly different between Meishan and Landrace in 5-months pigs according to their sex^42^. In addition to the different androgen levels, CNV could be suggested as another cause of differential expression of several CYPs. Because CYPs are also important in the drug metabolism of pigs, CNV of CYP should be considered when studying pigs as a model animal for drug metabolism.

There were NEW-specifically duplicated genes such as *EFHC1*, *ZWINT*, and *ROPN1L,* but little was revealed about their function in pig. Moreover, the number of NEW individuals here were only two, which was too few to suppose these genes play important role in evolution of NEW. In addition, Previous studies were not enough to investigate the functional impact of copy number variation of like-genes such as *MARCKSL1, HSBP1L1,* and *NIF3L1* in the pig. Furthermore, the copy number of *MARCKS* and *HSBP1* were not significantly variable in both ${V}_{ST}$ and the Kruskal–Wallis test. *MYO1H* had not been reported yet about their phenotype and genomic variation in *Sus scrofa*.

## Conclusions

In this study, we explored copy number variable genes of pig populations and estimated differentiation in copy number of genes on CNVRs between populations. Also, the CNV of Woori-Heukdon was firstly investigated, and it presented similarity of CNV among recently developed breeds and their paternal/maternal populations. Although this is one of the largest porcine CNV studies, the case of minor variants suggested the limitation of CNV calling using NGS read alignment with single reference genome. In further studies, we anticipate that additional high-quality genome assemblies representing various populations, and experimental validation would improve evolutionary insights on the CNV of pigs.

## Supplementary Information

Supplementary Legends.

Supplementary Figure S1.

Supplementary Tables.

## Data Availability

The newly generated sequences for 5 Korean Native Pigs, 5 Woori-Heukdon, and 5 Duroc individuals are available from Sequence read archive (SRA) with the Bioproject Accession Number PRJNA843521 (https://www.ncbi.nlm.nih.gov/bioproject/PRJNA843521). All other whole genome sequence data are available in NCBI SRA database (Table S1).

## Abbreviations

CNV: Copy number variation
SNP: Single nucleotide polymorphism
CNVR: Copy number variation region
AD: Asian domesticated pigs
AW: Asian wild boar
ED: European domesticated pigs
EW: European wild boar
NEW: Near Eastern wild boar
