# Supplementary material for: Population differentiated copy number variation between Eurasian wild boar and domesticated pig populations
Source: Sci Rep. 2023 Jan 20;13:1115. doi: 10.1038/s41598-022-22373-z (PMC9859782; doi:10.1038/s41598-022-22373-z)
Supplement: Supplementary file 1 — Supplementary Legends. [file 41598_2022_22373_MOESM1_ESM.docx]

**Supplementary figure and table legends**

**Figure S1. Hierarchical clustering tree**

For every individual, the absence or presence of CNVs in autosomal CNVRs was converted to a vector made of ‘0’s and ‘1’s. The hierarchical clustering was performed on these vectors representing each individual. The bootstrap value was shown under the edges of the clustering. The approximately unbiased (AU) and the bootstrap probability (BP) *p*-value were written in red and green letters on the edges after multiplied by 100.

**Table S1. Sample Information, alignment stats and summary of CNVs.**

**Table S2. Results of autosomal CNV calling using CNVnator and Lumpy.**

**Table S3. Chromosome-wise distribution of CNVs.**

**Table S4. Different distribution of chromosome-wise CNV between sexes.**

**Table S5. CNV distribution on p-arm and q-arm.**

**Table S6. Average lengthening and shortening of chromosomal length in each groups.**

**Table S7. Genes on CNVRs sorted by their genomic regions.**
