# Supplementary material for: Population differentiated copy number variation between Eurasian wild boar and domesticated pig populations
Source: Sci Rep. 2023 Jan 20;13:1115. doi: 10.1038/s41598-022-22373-z (PMC9859782; doi:10.1038/s41598-022-22373-z)
Supplement: Supplementary file 2 — Supplementary Figure S1. [file 41598_2022_22373_MOESM2_ESM.pdf]

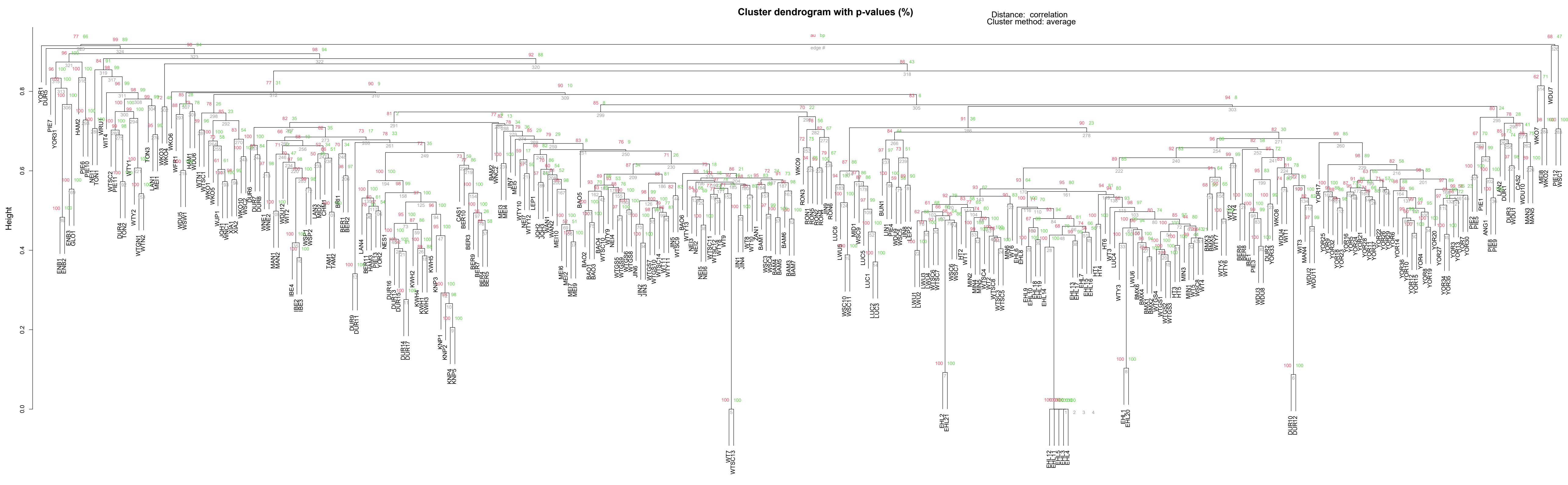
Cluster dendrogram with p-values (%)
Distance: correlation
Cluster method: average
au
bp
edge #
Height
0.0
0.2
0.4
0.6
0.8
